# Resection of synchronous bilateral multiple lung adenocarcinomas using virtual-assisted lung mapping

**DOI:** 10.1186/s40792-018-0441-4

**Published:** 2018-04-04

**Authors:** Naoko Imanishi, Shuichi Shinohara, Taiji Kuwata, Fumihiro Tanaka

**Affiliations:** Second Department of Surgery, University of Occupational and Environmental Health, Iseigaoka 1-1, Yahata-nishi-ku, Kitakyusyu, 807-8555 Japan

**Keywords:** Multiple lung cancer, Mapping, Sub-lobar resection

## Abstract

**Background:**

Virtual-assisted lung mapping (VAL-MAP) is a novel marking technique to assist sub-lobar resection of small hardly palpable lung tumors. Here, we present the first case of synchronous bilateral multiple lung adenocarcinomas that were successfully resected with VAL-MAP navigation.

**Case presentation:**

A 73-year-old female with multiple nodules (1 in the right upper lobe, 2 in the right lower lobe, 1 in the left upper lobe, and 1 in the left lower lobe) was referred. Complete resection was achieved with left lower lobectomy in combination with sub-lobar resections (wedge resection for a lesion in the left upper lobe, segmentectomy for a lesion in the right upper lobe, and complex segmentectomy for lesions in the right lower lobe) in which resection lines with securing adequate margins were determined with VAL-MAP navigation.

**Conclusions:**

VAL-MAP is useful in sub-lobar resections including complex segmentectomy for multiple lung adenocarcinomas.

## Background

Recent prevalence of lung cancer screening with computed tomography (CT) has contributed to early detection of multiple lung cancer [1], and 1227 patients with multiple lung cancer were operated during 2014 in Japan [2]. For such patients, sub-lobar resection may be preferable for preservation of sufficient pulmonary function after surgery, and exact identification and resection of each tumor with adequate resection margin is critical for its curability.

Virtual-assisted lung mapping (VAL-MAP) is a novel bronchoscopic marking technique to assist sub-lobar resections of small hardly palpable lung tumors [3, 4], and the feasibility was confirmed in a multi-institutional prospective study [5]. Here, we report the first case of bilateral multiple lung adenocarcinomas successfully resected by lobectomy in combination with sub-lobar resections (wedge resection, simple segmentectomy, and complex segmentectomy) with VAL-MAP navigation.

The VAL-MAP navigation was approved by the Ethics Committee of the University of Occupational and Environmental Health, Japan (H26–003), and was performed after written informed consent from the patient.

## Case presentation

**Fig. 1:**
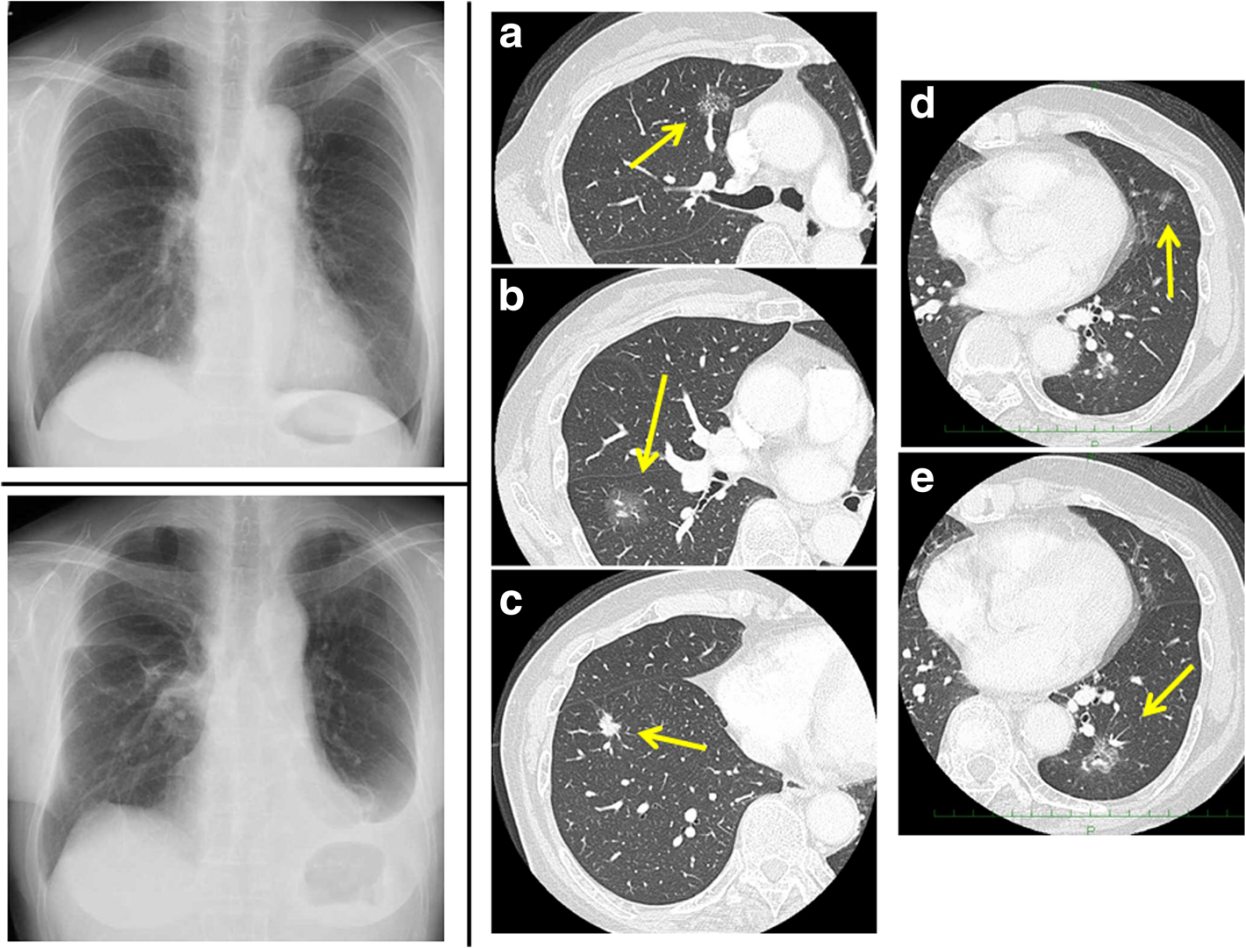
(Left, upper) Chest roentgenogram at presentation. (Right) Preoperative computed tomography (CT) showing bilateral lung nodules as follows: **a** part-solid ground-glass nodule (GGN, 23 mm) in the anterior segment (S3) of the right upper lobe, **b** pure GGN (24 mm) in the superior segment (S6) of the right lower lobe, **c** solid nodule (13 mm) in the anterior basal segment (S8) of the right lower lobe, **d** pure GGN (8 mm) in the lingular segment of the left upper lobe, and **e** part-solid GGN (35 mm) in the left lower lobe. (Left lower) Chest roentgenogram after two-staged bilateral operations

**Fig. 2:**
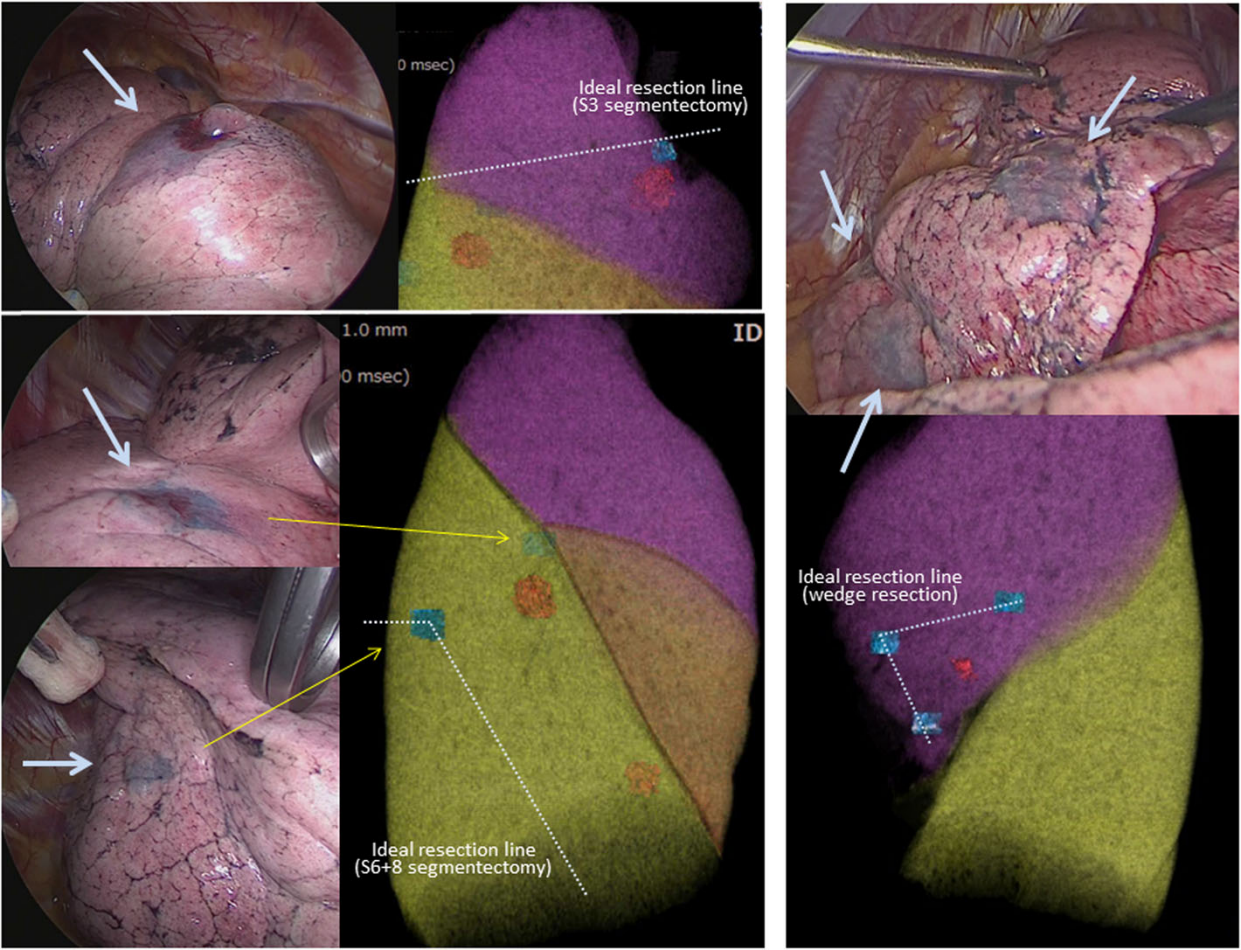
Three-dimensional images for planning of “virtual-assisted lung mapping (VAL-MAP)” indicating target nodules (red) and planned markings (blue) and thoracoscopic view of actual markings (blue) on the lung surface. (Left upper) A marking spot was designed to indicate an ideal resection line in S3 segmentectomy. (Left lower) A marking spot was designed to indicate an ideal resection line in complex segmentectomy of S6 plus S8, and another spot was designed to identify the target lesion (pure GGN) in S6. (Right) Three markings were designed to indicate an ideal resection line in wedge resection for the small pure GGN (8 mm) in the lingular segment

A 72-year-old female never-smoker was referred for resection of bilateral multiple lung nodules revealed on chest CT (Fig. 1). As each nodule was radiographically suspicious of primary lung adenocarcinoma without nodal or distant metastasis, curative-intent surgery consisting of left lower lobectomy for the largest nodule (35 mm) and sub-lobar resections with VAL-MAP navigation for the other nodules was planned. For VAL-MAP, virtual bronchoscopy and three-dimensional (3D) images were generated using a software (Bf-NAVI® [Cybernet Systems, Tokyo, Japan]) (Fig. 2). On the day before surgery, lung mapping was performed by injecting dye (1 ml of indigo carmine [Daiichi-Sankyo, Inc., Tokyo, Japan]) via standard flexible bronchoscopy into each target bronchus.

We achieved complete resection of all nodules with two-staged thoracoscopic operations. First, we performed right-sided operation consisting of S3 segmentectomy and complex segmentectomy of S6 plus S8 with VAL-MAP navigation. Marking spots were designed to navigate ideal resection lines in segmentectomies beyond anatomical inter-segmental planes with adequate resection margins. For example, two VAL-MAP markings, one indicating the place of S6 tumor and another indicating the “turning point” during lung stapling lines, were made for S6+S8 segmentectomy (Fig. 2). During thoracoscopic operation, all segmental arteries (A6 and A8), veins (V6 and V8), and bronchi (B6 and B8) were first ligated and dissected, and thereafter, segmentectomy was completed by stapling the lung along with planned resection lines as marked with VAL-MAP navigation (Fig. 2). Six weeks later, we performed left lower lobectomy in combination with wedge resection with VAL-MAP navigation (Fig. 2). The final pathological diagnosis was minimally invasive adenocarcinoma or the largest nodule and adenocarcinoma in situ for the other nodules.

### Discussion

We presented the first case of synchronous bilateral adenocarcinomas that were completely resected with lobectomy in combination with sub-lobar resections with VAL-MAP navigation. VAL-MAP is useful not only in wedge resection to identify small hardly palpable lung tumors with adequate resection margin but also in segmentectomy to determine resection lines with securing adequate margins. As compared with previous methods to determine resection margins in segmentectomy such as the usage of infiltration-deflation lines and injection of indocyanine green into selected pulmonary artery, VAL-MAP has an advantage of easy performing under thoracoscopic surgery without any special instrument. In addition, there is no need for lung inflation that may be easily performed by anesthetists and surgeons who are not familiar with infiltration-deflation techniques. More importantly, VAL-MAP has another advantage of capability to flexibly determine ideal resection lines beyond anatomical inter-segmental planes to provide adequate resection margins, as shown in the present case for complex segmentectomy of S6 plus S8 as well as for simple segmentectomy of S3. Despite these advantages, VAL-MAP navigation have several limitations such as difficulty in securing deep resection margins [3–5]. More sophisticated techniques should be developed to overcome these limitations.

## Conclusions

VAL-MAP is useful in sub-lobar resections for multiple lung adenocarcinomas.

## Abbreviation

VAL-MAP: Virtual-assisted lung mapping
